# Parental Knowledge on Thalassaemia and Factors Associated with Refusal to Screen Their Children

**DOI:** 10.21315/mjms2020.27.1.13

**Published:** 2020-02-27

**Authors:** Mohammad Azmi Che Mat, Lili Husniati Yaacob, Rosnani Zakaria

**Affiliations:** Department of Family Medicine, Universiti Sains Malaysia, Kubang Kerian, Kelantan, Malaysia

**Keywords:** thalassemia, knowledge, screening, school children

## Abstract

**Introduction:**

Acceptance of a screening programme for thalassemia is important in managing the disease and its associated complications. The objective of this study was to determine the knowledge of thalassemia and factors associated with thalassemia screening refusal among parents of secondary school children.

**Methods:**

This cross-sectional study was carried out from May 2017 to October 2017 among parents of fourth form students in three schools in Besut, Terengganu, Malaysia. Parents who are able to read and understand Malay and consented to the study were required to answer a validated questionnaire on their knowledge regarding thalassemia. They were also asked the reason for their acceptance or refusal of the thalassemia screening of their children.

**Results:**

In total, 273 participants took part in the study. The mean thalassemia knowledge score was 11.8 out of a maximum score of 21. Low knowledge scores (adjusted odds ratio [adj OR] 0.87; 95% confidence interval [CI]: 0.79, 0.95; *P* = 0.002) and female sex (adj OR 2.60; 95% CI: 1.04, 6.53; *P* = 0.040) had significant associations with parental thalassemia screening refusal. The main reason for screening refusal was that parents perceived that their children were not at risk for the disease since they did not have a family member with thalassemia.

**Conclusion:**

The mean thalassemia knowledge score among parents remains unsatisfactory. A high knowledge score is important since it is associated with parental acceptance of thalassemia screening for their children.

## Introduction

Thalassemia is a group of inherited genetic diseases characterised by a decrease in or the absence of the synthesis of the alpha or beta polypeptide chains that form the normal adult haemoglobin molecule (1). Globally, 5% of the world’s population are estimated to have a globin variant, while only 1.7% have the alpha or beta thalassemia trait (2). Thalassemia is the most common inherited disease in Malaysia, particularly alpha and beta thalassemia (3). As of June 2014, the Malaysian Thalassemia Registry had recorded as many as 6,056 patients. Among them, 4,394 patients had beta thalassemia or HbE beta thalassemia and as many as 3,000 of those patients required blood transfusion therapy (4).

Patients with thalassemia present along a spectrum ranging from nearly asymptomatic to severe anaemia requiring lifelong blood transfusions. In addition, they may also develop complications from the disease or its treatment, including bone changes, stunted growth, heart failure, delayed puberty and iron overload (5, 6).

The treatment options for thalassemia patients include bone marrow transplants and blood transfusion therapy, which put a huge economic burden on the Malaysian health system. The Malaysian government funds the cost of the therapy, including the provision of the chelation agent, subcutaneous desferioxamine (7). It is, therefore, crucial to strengthen the screening programme to ease the burdens on the patients and the government. Preventing the birth of babies with thalassemia major would allow the redistribution of funding to provide optimal care for existing thalassemia patients and alleviate their lifelong socioeconomic burden.

Thalassemia screening programmes have been implemented in multiple countries for many years. They differ by country in terms of mandatory versus voluntary screening and the timing of the testing at pre-conception versus in the early trimester of pregnancy. Screenings of 15–16-year-old school students, pre-marital screenings and screenings of the relatives of known carriers can effectively identify the beta thalassemia trait and control the disease (6). In Malaysia, the school thalassemia screening programme was started in 2016 and involves fourth form students. The aim of our study was to determine the knowledge of thalassemia and the factors associated with thalassemia screening refusal among parents of secondary school children.

## Methods

This was a cross-sectional study carried out from May 2017 to October 2017, which included 273 parents of fourth form students from three secondary schools in Besut, Terengganu, Malaysia.

### Sample Size Calculation

The estimated sample size was calculated based on a study conducted in Malaysia that reported the mean (standard deviation [SD]) knowledge score for the general population as 11.85 (4.03) (8). The sample size was calculated using a single mean formula with the precision set at 0.5 and a confidence interval (CI) of 95%. The minimum sample size was 250, and, after considering a 20% non-response rate, the calculated sample was 300 parents.

### Data Collection Procedures

As noted above, the study was conducted in Besut, Terengganu, Malaysia. The area was chosen because participation in thalassemia screening in the district in the previous year was low (just below 50%); out of those screened, 22.6% were suspected to be thalassemia carriers based on full blood count tests (9). In 2017, 20 secondary schools in the Besut district were selected for the thalassemia screening programme by the Besut District Health Office. Of those 20 schools, Sekolah Menengah Kuala Besut, Sekolah Agama Ittifakiah and Sekolah Menengah Kebangsaan Nasiruddin Shah were selected for the present study using simple random sampling. In each school, 100 students were selected by systematic random sampling based on the list of fourth form students. The ratio of systematic samplings was 1:2 to 1:3, according to the number of students in each school. Parents of the selected students who met the inclusion criteria were recruited as participants. The inclusion criteria were parents of fourth form students who are able to read and understand Malay. Those with severe psychiatric illness or mental retardation were excluded from the study.

This study was conducted in conjunction with the thalassemia screening programme carried out by the School Health Team from the Ministry of Health (MOH). The headmasters and teachers were briefed on the study and lists of fourth form students from each school were obtained from the school administrators. Sampling was done according to the sampling frame, which was used to identify the students. A brief discussion was held with these students to determine whether their parents fit the inclusion or exclusion criteria.

Invitation letters to join the study and come to the school on the screening day were given to the parents through the students two weeks prior to the event. Consent forms for the screening and pamphlets regarding thalassemia were also given to all parents of fourth form students two weeks prior to the event by MOH staff. The pamphlet contained general information on thalassemia, characteristics of thalassemia carriers and thalassemia patients, the targeted population for screening, the screening procedures, the benefits and risks of screening and the thalassemia inheritance pattern. The completed consent forms and feedback regarding participant attendance were collected by a teacher in charge prior to the screening day.

The data collection was performed during the thalassemia screening day. The 180 participants who were able to come to the thalassemia screening day were briefed regarding the study by the principal investigator. Another 120 participants, who consented for the study but were unable to attend the event, were briefed regarding the study through phone calls and separate appointments were arranged for them to answer the questionnaire. The inclusion and exclusion criteria were re-examined with the participants. For those that met the inclusion criteria, the questionnaires were distributed, and participants were required to complete them within half an hour. The submitted questionnaires were checked upon collection by the researcher for completion.

### Instruments

A self-administered questionnaire with three sections was used in this study. The first section primarily concerned sociodemographic data: age, sex, marital status, ethnicity, highest education level, occupation, monthly average household income and locality. The other three questions in this section were on the subjects’ awareness of thalassemia, presence of a family history of thalassemia carrier or thalassemia major and a history of a previous child screened for thalassemia.

The second part of the questionnaire consisted of 21 knowledge items on thalassemia, which were used by Wong et al. (8) in their study of awareness, perception and attitudes among the general population in Malaysia towards thalassemia and thalassemia prevention practices. The internal consistency (Cronbach’s alpha) of the original questionnaire for the knowledge domain was 0.86.

The questions underwent forward and backward translation from English to Bahasa Malaysia, and the content was validated by a panel of experts.

The questions covered four knowledge domains: i) general knowledge of thalassemia (five questions); ii) knowledge of thalassemia major (six questions); iii) knowledge of thalassemia carrier (eight questions); iv) knowledge of thalassemia prevention (two questions). A correct response for each question was scored as one, while incorrect or ‘don’t know’ responses were scored as zero. The total scores were obtained by adding the response scores, with a minimum possible score of zero and a maximum score of 21.

The third part of the questionnaire was on the subjects’ decision regarding the screening itself. They needed to state whether they agreed to have their child screened and to give reasons for their decision. Five reasons for acceptance and nine reasons for refusal were listed and the subjects could write in any other reasons if they chose. The five reasons of acceptance were: i) my child is at risk of having the thalassemia disease or being carriers, ii) my child wants to know his/her thalassemia status, iii) my child wants to participate in the screening programme, iv) my child wants to be screened and v) other. The nine reasons for refusal were: i) my child has been screened before, and the result was normal; ii) my child was screened, and the result was either thalassemia major or carrier; iii) I do not believe my child is at risk since there are no family members with thalassemia major or carrier; iv) I am afraid of the result; v) I am afraid of discrimination; vi) I believe my religion would disapprove of this screening; vii) my child refused to be screened; viii) my child choose to be screened at other time in the future; and ix) other.

### Statistical Analysis

Descriptive statistics were used to analyse the socio-demographic and knowledge scores, and the results were presented as means and frequencies. The association between the knowledge scores, socio-demographic factors and screening refusal was analysed using simple and multiple logistic regression. The significance level was set at a *P*-value < 0.05, with a 95% CI. All analyses were performed with the Statistical Package for Social Sciences (SPSS), version 24.

## Results

A total of 300 parents of fourth form students were recruited for the present study. One was excluded because he/she did not fulfil the eligibility criteria (case report form [CRF] answered by sibling). From the 299 remaining subjects, nine did not grant the written informed consent to join the study, and 17 did not fully complete the questionnaire. Therefore, the data were analysed for 273 subjects with a response rate of 91.3%.

Table 1 describes the characteristics of all participants. The majority were Malay, married and from a rural area. Most of them had up to a secondary school education and were either unemployed or unskilled workers.

The mean and median knowledge scores were 11.8 (SD 4.91) and 13 (interquartile range [IQR] 7), respectively. Table 2 describes the percentage of subjects who answered each knowledge item correctly and incorrectly. Regarding general knowledge on thalassemia, most of them knew that thalassemia is an inherited disease, which can be detected through a blood test but not a urine test. Furthermore, 71.4% correctly answered that a person with thalassemia can be either a carrier or have thalassemia major, but only 53.1% knew that thalassemia cases are classified into α and β groups.

In terms of knowledge regarding thalassemia major, the majority (71.4%) knew that these individuals can lead normal and healthy lives with appropriate treatment and have longer life expectancies (61.2%). Less than half (48.7%) recognised that individuals with thalassemia major need regular blood transfusions throughout their lives and knew that thalassemia major can be cured by a bone marrow transplant (34.4%). Concerning thalassemia carriers, 57.5% knew that carriers are asymptomatic and 50.9% knew they can play normal roles in society. However, 57.5% incorrectly believed that carriers show signs of anaemia, and only a small percentage of the subjects (32.2%) understood that thalassemia carriers will not develop thalassemia major.

In the present study, only 25 subjects (9.2%) refused to give consent for the thalassemia screening. The biggest reason for refusal was that they believed their child was not at risk (46%). The next most popular reason was that the child refused to be screened (38%), followed by fear of the result (8%) and other (8%).

Table 3 shows that age, sex and knowledge scores on thalassaemia, marital status, tertiary education level, occupation, locality and screening of previous children are significant associated factors (*P* < 0.25) for screening refusal for thalassemia using simple logistic regression analysis. All statistically significant and clinically significant variables were included in multiple logistic regression analysis.

In the analysis for multicollinearity and interaction, there was an interaction between knowledge score and sex; however, it was not highly significant (*P*-value = 0.029). Further analysis showed that the OR (95% CI) for female and low knowledge interaction was 0.04 (0.004, 0.309). This means that females with low knowledge scores were 0.04 more likely to refuse screening (*P =* 0.002) than males with high knowledge scores. It also means that screening refusal was modified by both gender and knowledge level. In term of model fitness, the overall correctly classified percentage was 91.6%, which is good because it was above 80%. The area under the curve was 78.1%, which means that it is useful for discrimination. The Hosmer-Lemeshow test with a *P*-value of 0.348 (*P*-value > 0.05) indicated that the model was fit.

Therefore, associated factors for refusal were knowledge score and gender. A person with a one point increase in his/her total knowledge score was 15% less likely to refuse screening for his/her child (95% CI: 0.79, 0.95; *P =* 0.002) and women were 2.6 times more likely to refuse thalassemia screening for their children (95% CI: 1.04, 6.53; *P =* 0.040) (Table 4).

## Discussion

In this study, the mean thalassemia knowledge score among the subjects was 11.8 out of a maximum score of 21. This result is similar to that of another study, which used the same questionnaire and was conducted nearly 10 years ago (8). However, the previous study involved multi-ethnic groups, multiple localities in Malaysia and included younger age groups. The findings in the present study indicate that there has not been much improvement in the knowledge of thalassemia in the near decade since the previous study was carried out. This raises the issue of whether existing education programmes on thalassemia are effective at increasing knowledge of the disease among the population. Most parents did have general knowledge on thalassemia, especially that it is inherited from parent to child and can be easily detected through blood test. In addition, almost three-quarters of parents knew that individuals with thalassemia can be divided into either patients (thalassemia major) or carriers. This proportion is higher than the one found in 2009 (8).

In general, the knowledge regarding thalassemia carriers was still inadequate. One fifth of the parents wrongly believed that the life expectancy of thalassemia carriers is short, and a quarter of them wrongly thought that carriers will develop thalassemia major. Parental understanding regarding the manifestation of thalassemia and the role of gene inheritance is important because it affects decision making regarding screening. Knowledge deficiency in these areas may result in limitations in receiving optimal healthcare in regard to carrier testing (10).

Regarding thalassemia major, that majority of parents understood that appropriate treatment is beneficial for prolonging patients’ life expectancies and enabling them to live normally and healthily. This knowledge is important since people are more likely to seek medical treatment and participate in screenings if they know that effective treatment is available for them. However, less than half of the parents were aware that individuals with thalassemia major need to receive blood transfusion regularly since their red blood cells rupture easily. This number is slightly less than the one in the 2009 study (8).

The present study also found that parents with higher knowledge scores were less likely to refuse screening for their children, but female parents were more likely to refuse screening for their children. Currently, there is limited data on the association between knowledge and parental decisions to screen children for haemoglobinopathies. However, multiple studies have been done on other inherited diseases, such as cystic fibrosis. A systematic review of cystic fibrosis concluded that knowledge deficiency is one of the factors of screening refusal (11). Three other studies revealed that having more knowledge on the inherited disease was associated with acceptance of the disease screening (12–14).

The importance of having high knowledge and participation levels in screening for thalassemia in other groups has been described by a few studies. Almost half of the participants in a study in Iran believed that increasing knowledge was the most vital step to preventing thalassemia, along with genetic counselling and premarital screening (15). Thalassemia education in Cambodia, even in populations with low education levels, was shown to increase knowledge and promote positive attitudes towards screening (16).

The present finding on the association between parental sex and screening refusal needs to be carefully considered. For example, even though the mother completed the questionnaire, the decision not to screen could have been discussed and decided by both parents and the child. Furthermore, one of the reasons for refusal was the child’s own refusal to be screened. Therefore, even though the mother declined to give consent, it does not necessarily mean that participant’s sex had any impact on the refusal.

Having said that, Al Farsi et al. (17) revealed that the female sex was associated with an unwillingness to complete premarital screenings in Oman, which is consistent with the present study’s finding. However, the authors noted that their study had a relatively small sample size, which could have affected its statistical significance (17). Meanwhile, multiple studies on premarital screenings of other diseases found that, generally, both males and females had favourable attitude towards it. Ganczak et al. (18) found that unmarried males had negative attitudes towards screening and suggested that education should be focused on in this group. Presumptively, parents should be more receptive towards the screening their children regardless of their sex. It would be interesting to retrospectively look at the consent forms of previous screenings and study whether parental sex significantly predicted the participation of their children in screenings for inherited diseases.

Even though this study had a low refusal rate for the thalassemia screening (9.2%), it is interesting to discuss the reasons for the refusals. The most common of parental refusal was that they believed their children were not at risk since there was absence of a family member with thalassemia major or carrier. Less than half of the unmarried participants in a local study indicated their unwillingness to be screened for similar reasons (8). Likewise, a recent study demonstrated that a number of Omanis did not undergo the screening test for similar reasons (17). In addition, parents would probably be more accepting of the test if they were worried that their children could have the disease. Another reason for refusal, especially by mothers, was the fear of stigmatisation. Findings from previous studies reported that unfavourable responses toward screenings were due to the fear of being stigmatised and concerns that the carrier status could negatively impact future marriage prospects (10, 19).

The present study has several limitations. First, the study used a cross-sectional method in which causality and temporal relationships cannot be inferred from the results. Second, since the majority of the participants were Malay, the sample did not reflect the views of the multi-ethnic and multi-religious population in Malaysia. Third, although the study looked at thalassemia screening refusal, only 9.2% of subjects refused to screen their children; this low percentage might limit the study by not giving an accurate portrayal of the reasons for screening refusal.

A few recommendations can made based on the results of this study. Our study showed there is still a knowledge deficiency, especially regarding thalassemia carriers. A more effective education programme needs to be created, especially for use within the school health programme, since this is the best avenue for thalassemia education. Furthermore, the current two-page pamphlet on thalassemia might not contain enough clear information for parents to fully understand the disease and, hence, make an informed decision on screening.

## Conclusion

The mean thalassemia knowledge scores among parents are still unsatisfactory. As found in this study, having a high knowledge score is important since it is associated with parental acceptance of thalassemia screening for their children. The findings from this study can be used to improve the screening programme and help develop more effective public health education.

## Figures and Tables

**Table 1:** Characteristics of study participants (*n* = 273)

Variables	Median (IQR)	*n* (%)
Age (years)	49.0 (9.0)	
Incomes (RM)	900 (1200)	
Sex		
Male		153 (56.0)
Female		120 (44.0)
Marital Status		
Married		256 (93.8)
Divorced		17 (6.2)
Education Level		
Primary school		31 (11.4)
Secondary school		199 (72.9)
Tertiary level		43 (15.7)
Occupation		
Professional & skilled worker		94 (34.4)
Unskilled worker & unemployed		179 (65.6)
Locality		
Urban		40 (14.7)
Rural		233 (85.3)
Family history of thalassaemia		
Yes		14 (5.1)
No		259 (94.9)
Thalassaemia screening of previous child		
Yes		94 (30.8)
No		179 (69.2)

**Table 2:** Participants’ responses on knowledge on thalassaemia

No	Items	Correctly answered *n* (%)	Incorrectly answered *n* (%)	Don’t know *n* (%)
1	Thalassaemia is a hereditary disease	239 (87.5)	7 (2.6)	27 (9.9)
2	A person can be a thalassaemia carrier or have thalassaemia major?	195 (71.4)	10 (3.7)	68 (24.9)
3	Thalassaemia can be divided into two groups—alpha or beta thalassaemia	145 (53.1)	16 (5.9)	112 (41)
4	Urine test can be performed to determine if a person has thalassaemia	195 (71.4)	9 (3.3)	69 (25.3)
5	Blood testing can be performed to determine if a person has thalassaemia	249 (91.2)	4 (1.5)	20 (7.3)
6	The life span of a thalassaemia major patient is shortened if he/she does NOT receive appropriate treatment	167 (61.2)	16 (5.9)	90 (33.0)
7	Red blood cells of thalassaemia major patients break down easily and cause anemia	127 (46.5)	21 (7.7)	125 (45.8)
8	Individuals with thalassaemia major require regular blood transfusions throughout life	133 (48.7)	31 (11.4)	109 (39.9)
9	All thalassaemia major individuals are mentally retarded	152 (55.7)	17 (6.2)	104 (38.1)
10	Individuals with thalassaemia major can lead normal and healthy lives with appropriate treatment	195 (71.4)	14 (5.1)	64 (23.4)
11	Individuals with thalassaemia major can be cured with bone marrow transplant	94 (34.4)	42 (15.4)	137 (50.2)
12	The lifespan of thalassaemia carrier is shortened	156 (57.1)	59 (21.6)	58 (21.2)
13	Thalassaemia carriers appear healthy and show no symptoms	157 (57.5)	46 (16.8)	70 (25.6)
14	A thalassaemia carrier shows signs of anemia (pale), tiredness, no appetite and prone to infections	55 (20.1)	157 (57.5)	61 (22.3)
15	A thalassaemia carrier may become a Thalassaemia major	88 (32.2)	66 (24.2)	119 (43.6)
16	A thalassaemia carrier cannot play a normal role in society with regards to working and having a family and needs treatment for the disease	139 (50.9)	60 (22.0)	74 (27.1)
17	Children born to both parents who are thalassaemia carriers will be at risk of having thalassaemia major	174 (63.7)	28 (10.3)	71 (26.0)
18	Children born to either one parent who is a thalassaemia carrier will be at risk of having thalassaemia major	80 (29.3)	98 (35.9)	95 (34.8)
19	Couples where both are thalassaemia carriers, will certainly give birth to thalassaemia major children, they will not conceive any healthy children	65 (23.8)	128 (46.9)	80 (29.3)
20	The partner of a thalassaemia carrier should undergo blood tests	241 (88.3)	1 (0.4)	31 (11.4)
21	Prenatal diagnosis for high risk pregnancies (both partners are thalassaemia carriers) are necessary	180 (65.9)	19 (7.0)	74 (27.1)

**Table 3:** Associated factors of parental refusal for thalassaemia screening using simple logistic regression

Variable	Crude odds ratio (OR) (95%CI)	Wald statistic	*P*-value
Age (years)	0.96 (0.89, 1.03)	1.59	0.206
Income (RM/month)	1.00 (1.00, 1.00)	0.09	0.755
Knowledge score on thalassemia	0.85 (0.78, 0.92)	15.34	0.000
Sex			
Man	1		-
Woman	2.11 (0.88, 5.07)	2.81	0.093
Marital Status			
Married	1		-
Divorced	2.52 (0.67, 9.54)	1.88	0.171
Education level			
Primary school	1		-
Secondary school	0.63 (0.20, 2.02)	0.61	0.436
Tertiary level	0.33 (0.06, 1.92)	1.52	0.217
Occupation			
Professional & skilled worker	1		-
Unskilled worker & unemployed	1.99 (0.72, 5.54)	1.74	0.188
Locality			
Urban	1		-
Rural	4.07 (0.53, 31.05)	1.83	0.176
Awareness on thalassaemia			
Yes	1		-
No	1.90 (0.60, 6.01)	1.18	0.278
Family history of thalassaemia			
Yes	1		-
No	0.53 (0.11, 2.52)	0.64	0.425
Screening of previous child for thalassaemia			
Yes	1		-
No	10.93 (1.45, 82.53)	5.38	0.02

**Table4:** factors of parental refusal for thalassaemia screening using multiple logistic Significant regression

Variable	Crude OR (95% CI )	adj OR (95% CI)	Wald statistic	*P*-value
Female sex	2.11 (0.88, 5.07)	2.60 (1.04, 6.53)	4.22	0.040
Knowledge score on thalassemia	0.85 (0.78, 0.92)	0.87 (0.79, 0.95)	9.66	0.002

Note: constant −3.134
